# Mathematical modelling of bacterial resistance to multiple antibiotics and immune system response

**DOI:** 10.1186/s40064-016-2017-8

**Published:** 2016-04-05

**Authors:** Bahatdin Daşbaşı, İlhan Öztürk

**Affiliations:** Department of Computer Technologies, Kangal Vocational High-Schools, Cumhuriyet University, 58840 Sivas, Turkey; Department of Mathematics, Faculty of Sciences, Erciyes University, 38039 Kayseri, Turkey

**Keywords:** Ordinary differential equations systems, Equilibrium points, Immune system, Bacterial resistance, Antibiotics, 34K20, 92C50, 92D25

## Abstract

Resistance of developed bacteria to antibiotic treatment is a very important issue, because introduction of any new antibiotic is after a little while followed by the formation of resistant bacterial isolates in the clinic. The significant increase in clinical resistance to antibiotics is a troubling situation especially in nosocomial infections, where already defenseless patients can be unsuccessful to respond to treatment, causing even greater health issue. Nosocomial infections can be identified as those happening within 2 days of hospital acceptance, 3 days of discharge or 1 month of an operation. They influence 1 out of 10 patients admitted to hospital. Annually, this outcomes in 5000 deaths only in UK with a cost to the National Health Service of a billion pounds. Despite these problems, antibiotic therapy is still the most common method used to treat bacterial infections. On the other hand, it is often mentioned that immune system plays a major role in the progress of infections. In this context, we proposed a mathematical model defining population dynamics of both the specific immune cells produced according to the properties of bacteria by host and the bacteria exposed to multiple antibiotics synchronically, presuming that resistance is gained through mutations due to exposure to antibiotic. Qualitative analysis found out infection-free equilibrium point and other equilibrium points where resistant bacteria and immune system cells exist, only resistant bacteria exists and sensitive bacteria, resistant bacteria and immune system cells exist. As a result of this analysis, our model highlights the fact that when an individual’s immune system weakens, he/she suffers more from the bacterial infections which are believed to have been confined or terminated. Also, these results was supported by numerical simulations.

## Background

Infections have been the leading cause of most diseases in the history of mankind (Mondragón et al. 2014). Especially bacterial infections are more prevailing among these. The most common procedure known to fight bacterial infection is through antibiotic therapy applied to individuals. The expression of resistance to antimicrobial agents in this therapy is both the logical and indispensable outcome of using these agents to treat human infections (Mahmoud and Rice 1999; Ternent et al. 2014).

Resistance developed by the bacteria against antibiotics is described as the talent of bacteria to resist the effects of antibiotics generated either to eradicate or control them (Arya 2007). The release of each new class of antibiotics for treatment, shortly after, has been followed by the emergence of new strains of bacteria which are resistant to this class (Butler and Buss 2006; Clatworthy et al. 2007; Lewis 2013). In this respect, developing new treatment strategies for bacterial infections is very important (Mondragón et al. 2014).

According to its properties, antibiotics has the bacteriostatic action to stop the growth of bacteria and bactericidal action to wipe out the bacteria. However, the distinction between these properties is not explicit, as it depends on the drug concentration used and the type and the growth stage of bacteria (Zhang 2009). Hence, multiple antibiotic therapy is a more appropriate form of treatment.

In fact, the bacterial infection is a complicated process for both the infectious bacteria and the host (Carvalho et al. 2012). It is suggested that a significant role in the progress of infections is basically played by the immune system (Linares and Martinez 2005). The immune system is expressed as a system of biological structures and processes in an organism protecting the body from likely harmful substances via recognizing and responding to antigens (Alberts et al. 2002). In this context, the reactions of different hosts against the same infection can be different due to immune system’s response given by host.

In light of the above, dynamics of relations among antibiotics, immune system’s cells and bacteria are important to understand the nature of the infection.

Mathematical models are one of the significant tools used in both analyzing the spread of infectious diseases in a population of individuals (Hethcote 2000; Singer 1984), and predicting the timing and expansion of infection and possible reinfection processes in an individual (Mohtashemi and Levins 2001). While the former is usually used for planning, prevention and control scenarios, the latter can be active in therapy/intervention programs for treating the individuals exposed to the particular pathogen. In this respect, understanding and anticipating the time of occurrence and magnitude of the maximum load of the bacteria and immune system cells by mathematical modelling can be crucial in selecting effective intervention strategies (Whitman and Ashrafiuon 2006).

Consequently, results on reproduction of sensitive and resistant bacteria to antibiotics are obtained in Austin et al. (1997), Bonten et al (2001), Esteva et al. (2011), Wiesch et al. (2011), Zhang (2009); definitions of factors responsible for resistance prevalence are studied in Austin and Anderson (1999), Linares and Martinez (2005), Opatowski et al. (2011), Rodrigues et al. (2007); bacteria behavior under different antibiotic treatments is examined in Alavez et al. (2006), Bonhoeffer et al. (1997), Bootsma et al. (2012), D’Agata et al. (2007), Sun et al. (2010); optimization results and design of control measures are investigated in Bonten et al. (2001), Haber et al. (2010), Massad et al. (2008), Sotto and Lavigne (2012); biological cost and persistence of antibiotic resistance are analyzed in Andersson et al. (2001), Andersson and Levin (1999), Antia et al. (1996), Johnson and Levin (2013), Mondragón et al. (2014); dynamics between pathogens and immune response are given in André and Gandon (2006), Carvalho et al. (2012), D’Onofrio (2005), Gilchrist and Coombs (2006), Gilchrist and Sasaki (2002), Kostova (2007), Mohtashemi and Levins (2001), Nowak and May (2000), Whitman and Ashrafiuon (2006), respectively.

In this study, we have constructed a continuous time model considering the immune response of the host and the basic mechanisms of bacterial resistance to antibiotics. Our aim is to find specific parameters determining the change in the concentrations of the immune system’s cells produced in host to fight these and the sensitive sub-populations and resistant sub-populations that has either arisen through random mutation and clonal enlargement or through cross-contamination in a special infection and under a appropriate treatment regimen.

## Model formulation

When the emergence of resistant bacteria to antibiotic has modelled, there are the two basic aspects generally considered. These are within-host models and within-hospital compartmental models. Within-hospital compartmental models are generally *SIR* models in epidemiology, considering especially how infections will expand throughout the hospital (Ternent et al. 2014). These models are useful to develop strategies to prevent the spread of resistant individuals to antibiotic in hospital.

Current mathematical models focusing on the fact that it is within the host that resistance develops, aim to study how antibiotic treatment strategies can affect, and in addition, how the occurrence of antibiotic resistance can be prevented (Alberts et al. 2002; Hethcote 2000). In such models, the effect of immune cells generated by the host under the pressure of the bacteria are frequently either neglected or assumed to be at a constant rate. We built up the mathematical model including cell-mediated immune response. In addition, treatment regimens involving multiple antibiotic have been used in most bacterial infections due to bacterial resistant. In this sense, besides the interaction of bacterial-immune response, to investigate the effects of multiple drug therapy is biological more meaningful.

We analyzed the concentrations of the immune cells and bacteria in an individual receiving a cocktail of multi-drug treatment against bacteria via mathematical modelling. Let us denote by *S*(*t*) and *R*(*t*) the population sizes of sensitive and resistant bacteria against multiple antibiotics at time *t*, respectively, by *B*(*t*) population sizes of immune cells at time *t*, and by *A*_*i*_(*t*) the concentration of the *i*-th antibiotic, for *i* = 1, 2, …, *n* at time *t*. The parameters used in the model are as follows.

We assume that bacteria follow a logistic growth with carrying capacity *T*. Let *β*_*S*_ and (1 − *c*)*β*_*S*_ the birth rate of sensitive and resistant bacteria, respectively. Specific mutations arising from resistance to chemical control frequently have an inherent fitness cost which may be resulted through reduced reproductive capacity or competitive ability (Alavez et al. 2006). We evaluate fitness cost as a reduction on the reproduction rate of the resistant strain, therefore 0 < *c* < 1. Likewise, we have supposed that using a logistic style term, immune cells are recruited to the region of infection at a rate *k* and carrying capacity of these is as *ω* fold of amount of present bacteria. This is biologically very significant in terms of proliferation of specific immune cells. Also, these interacts can be expressed as a generalised model of a local bacterial infection, such as a urinary tract, tuberculosis or wound infection.

During the administration of the *i* th antibiotic, a number of resistant bacteria to it can be showed up due to mutations of exposed sensitive bacteria to such antibiotic, we model this situation by the term $\overline{{\alpha_{i} }} A_{i} S$ where $\overline{{\alpha_{i} }}$ is the mutation rate of sensitive bacteria due to exposure to *i* th antibiotic. Sensitive and resistant bacteria have per capita death rates by response of immune cells and this rates is *λ*. Sensitive bacteria also die due to the action of the antibiotics, and we have assumed that this situation in model is by the term $\overline{{d_{i} }} A_{i} S$, where $\overline{{d_{i} }}$ is the death rate of sensitive bacteria due to exposure to *i*-th antibiotic. Lastly, the *i*-th antibiotic concentration is supplied at a constant rate *δ*_*i*_, and is taken up at a constant per capita rate *μ*_*i*_. Under the assumptions a fore mentioned, we obtain the following system of (*n* + 3) ODE’s:

1 $$\begin{aligned} \frac{{dS}}{{dt}} & = \beta _{S} S\left( {1 - \frac{{S + R}}{T}} \right) - \lambda SB - S\sum\limits_{{i = 1}}^{n} {\overline{{\alpha _{i} }} } A_{i} - S\sum\limits_{{i = 1}}^{n} {\overline{{d_{i} }} } A_{i} \\ \frac{{dR}}{{dt}} & = \left( {1 - c} \right)\beta _{S} R\left( {1 - \frac{{S + R}}{T}} \right) - \lambda RB + S\sum\limits_{{i = 1}}^{n} {\overline{{\alpha _{i} }} } A_{i} \\ \frac{{dB}}{{dt}} & = kB\left( {1 - \frac{B}{{\omega \left( {S + R} \right)}}} \right) \\ \frac{{dA_{i} }}{{dt}} & = \delta _{i} - \mu _{i} A_{i} ,\quad i = 1,2, \ldots ,n. \\ \end{aligned}$$

where

2 $$\beta_{S} ,c,\lambda ,T,k,\omega ,\overline{{\alpha_{i} }} ,\overline{{d_{i} }} ,\delta_{i} ,\mu_{i} > 0 \quad {\text{for }}i = 1,2, \ldots ,n$$

To reduce the number of parameters, the variables are changed as follows;

$$s = \frac{S}{T},\;r = \frac{R}{T},\;b = \frac{B}{\omega T},\;a_{i} = \frac{{A_{i} }}{{\frac{{\delta_{i} }}{{\mu_{i} }}}}.$$

With the new variables, the normalized system is given as;

3 $$\begin{aligned} \frac{ds}{dt} &= \beta_{S} s\left( {1 - \left( {s + r} \right)} \right) - \eta sb - s\mathop \sum \limits_{i = 1}^{n} \left( {\alpha_{i} + d_{i} } \right)a_{i} \\ \frac{dr}{dt} &= \beta_{R} r\left( {1 - \left( {s + r} \right)} \right) - \eta rb + s\mathop \sum \limits_{i = 1}^{n} \alpha_{i} a_{i} \\ \frac{db}{dt} &= kb\left( {1 - \frac{b}{s + r}} \right) \\ \frac{{da_{i} }}{dt} &= \mu_{i} \left( {1 - a_{i} } \right)\quad {\text{for}}\;i = 1,2, \ldots ,n\\ \end{aligned}$$

where $\alpha_{i} = \bar{\alpha }_{i} \left( {\frac{{\delta_{i} }}{{\mu_{i} }}} \right),\;d_{i} = \bar{d}_{i} \left( {\frac{{\delta_{i} }}{{\mu_{i} }}} \right)$, $\beta_{R} = \left( {1 - c} \right)\beta_{S}$ and $\eta = \lambda \omega T$. The biologically studied region is given by the set

4 $$\varOmega = \left\{ {\left( {s,r,b,a_{1} , \ldots ,a_{n} } \right) \in R^{n + 3} : 0 \le s,r, 0 \le b \le s + r \le 1, 0 \le a_{i} \le 1, i = 1, \ldots ,n} \right\}.$$

The following proposition ensures that system (3) is well posed in the sense that the solutions with positive initial conditions started in *Ω* remain in this region for all *t* ≥ 0, and so, this solutions of system (3) have biological meaning.

### **Proposition 1**

*The regionΩ defined in* (4)* is positively invariant with respect to the system* (3).

### *Proof*

By adding the first two equations of the system (3),

5 $$\frac{ds}{dt} + \frac{dr}{dt} = \left( {\beta_{S} s + \beta_{R} r} \right)\left( {1 - \left( {s + r} \right)} \right) - \eta b\left( {s + r} \right) - s\mathop \sum \limits_{i = 1}^{n} d_{i} a_{i}$$

is obtained. Considering the region *Ω*, we have the following inequality;

6 $$\frac{{d\left( {s + r} \right)}}{dt} \le \beta_{S} \left( {s + r} \right)\left( {1 - \left( {s + r} \right)} \right).$$

By the solution according to (*s* + *r*) of inequality (6), it follows that 0 ≤ *s* + *r* ≤ 1 for all *t* ≥ 0. Furthermore, the solutions of the last *n* equations of system (3) are

7 $$a_{i} \left( t \right) = 1 + \left( { - 1 + a_{i} \left( 0 \right)} \right)e^{{ - \mu_{i} t}} \quad {\text{for}}\, i = 1,2, \ldots ,n .$$

where initial conditions satisfy 0 ≤ *a*_*i*_(0) ≤ 1 for *i* = 1, 2, …, *n*. Lastly, let $0 \le s + r = u\left( {\text{constant}} \right) \le 1$. Then the solution of third equation in system (3) is

8 $$b = \frac{u}{{1 + e^{ - kt - b\left( 0 \right)} }}$$

where initial conditions satisfy 0 < *b*(0) ≤ *s*(0) + *r*(0). From (8), it is obtained that 0 ≤ *b* ≤ *s* + *r* ≤ 1.

Hence, the vector field of system (3) restricted to the boundary of *Ω* does not include a point at the exterior of it. In this context, solutions starting in $\varOmega^{ + }$ remain in the region *Ω* for all *t* ≥ 0.

## Qualitative analysis of system (3)

We have examined the existence and stability of equilibria of system (3).

### **Proposition 2**

*Let*

9 $$\frac{{\beta_{S} - \mathop \sum \nolimits_{i = 1}^{n} \left( {\alpha_{i} + d_{i} } \right)}}{{\beta_{S} + \eta }} = A,\quad\frac{{\beta_{R} }}{{\beta_{R} + \eta }} = B,\quad\frac{{\mathop \sum \nolimits_{i = 1}^{n} \alpha_{i} }}{{\beta_{R} + \eta }} = C.$$

*We accept that the general expressions of the system’s equilibria show as*$E_{j} = \left( {\bar{s},\bar{r},\bar{b},\overline{{a_{i} }} } \right)$* for*$i = 1,2, \ldots ,n$* andj* = 0, 1, 2, 3.* Then, system* (3)* always hasE*_0_ = (0, 0, 0, 1, 1, …, 1) (*namely, the infection-free equilibrium point*), *E*_1_ = (0, 1, 0, 1, 1, …, 1), *E*_2_ = (0, *B*, *B*, 1, 1, …, 1)* contained inΩ*.* WhenA* > *B*,* in addition toE*_0_, *E*_1_* andE*_2_,* there exists a fourth the equilibrium point,*$E_{3} = \left( {A\frac{A - B}{A - B + C},A\frac{C}{A - B + C},A,1,1, \ldots ,1} \right)$,* inΩ*.

### *Proof*

In (9), it is clear that

10 $$B,C > 0$$

The equilibria of system (3) are given by the solutions of the system of following algebraic equations;

11 $$\begin{aligned} &\beta_{S} s\left( {1 - \left( {s + r} \right)} \right) - \eta sb - s\mathop \sum \limits_{i = 1}^{n} \left( {\alpha_{i} + d_{i} } \right)a_{i} = 0 \\ &\beta_{R} r\left( {1 - \left( {s + r} \right)} \right) - \eta rb + s\mathop \sum \limits_{i = 1}^{n} \alpha_{i} a_{i} = 0 \\ &kb\left( {1 - \frac{b}{s + r}} \right) = 0 \hfill \\ &\mu_{i} \left( {1 - a_{i} } \right) = 0\quad {\text{for}}\;i = 1,2, \ldots ,n. \\ \end{aligned}$$

From the last *n* equation of system (11), we have *a*_*i*_ = 1 for *i* = 1, 2, …, *n*. Consequently, the system (11) turns into following system;

12 $$\begin{aligned} &\beta_{S} s\left( {1 - \left( {s + r} \right)} \right) - \eta sb - s\mathop \sum \limits_{i = 1}^{n} \left( {\alpha_{i} + d_{i} } \right) = 0 \hfill \\ &\beta_{R} r\left( {1 - \left( {s + r} \right)} \right) - \eta rb + s\mathop \sum \limits_{i = 1}^{n} \alpha_{i} = 0. \\ &kb\left( {1 - \frac{b}{s + r}} \right) = 0. \\ \end{aligned}$$

From (12), it is obtained that either $\bar{b} = 0$ or $\bar{b} = \bar{s} + \bar{r}$.

- When $\bar{b} = 0$, we have that the equilibrium points are $E_{0} = \left( {0,0,0,1, \ldots ,1} \right)$, $E_{1} = \left( {0,1,0,1, \ldots ,1} \right)$ and
  $$E^{\imath } = \left( { - \frac{{\beta_{S} - \mathop \sum \nolimits_{i = 1}^{n} \left( {\alpha_{i} + d_{i} } \right)}}{{\beta_{S} \mathop \sum \nolimits_{i = 1}^{n} \alpha_{i} - \beta_{R} \mathop \sum \nolimits_{i = 1}^{n} \left( {\alpha_{i} + d_{i} } \right)}}\frac{{\beta_{R} }}{{\beta_{S} }}\mathop \sum \limits_{i = 1}^{n} \left( {\alpha_{i} + d_{i} } \right),\frac{{\beta_{S} - \mathop \sum \nolimits_{i = 1}^{n} \left( {\alpha_{i} + d_{i} } \right)}}{{\beta_{S} \mathop \sum \nolimits_{i = 1}^{n} \alpha_{i} - \beta_{R} \mathop \sum \nolimits_{i = 1}^{n} \left( {\alpha_{i} + d_{i} } \right)}}\mathop \sum \limits_{i = 1}^{n} \alpha_{i} , 0,1, \ldots ,1} \right)$$
  Although the equilibrium points *E*_0_ and *E*_1_ always exist in *Ω*, the equilibrium point *E*^*I*^ where signs of $\bar{s}$ and $\bar{r}$ are opposite, is biologically meaningless. Therefore, *E*^*I*^ is not in *Ω*.
- In case of $\bar{b} = \bar{s} + \bar{r}$, it is obtained the equilibrium points as following;
  $$E_{2} = \left( {0,\frac{{\beta_{R} }}{{\beta_{R} + \eta }},\frac{{\beta_{R} }}{{\beta_{R} + \eta }},1, \ldots ,1} \right)$$ and $$E_{3} = \left( {\bar{b}\frac{{\beta_{R} - \bar{b}\left( {\beta_{R} + \eta } \right)}}{{\left( {\beta_{R} - \bar{b}\left( {\beta_{R} + \eta } \right)} \right) - \mathop \sum \nolimits_{i = 1}^{n} \alpha_{i} }}, - \bar{b}\frac{{\mathop \sum \nolimits_{i = 1}^{n} \alpha_{i} }}{{\left( {\beta_{R} - \bar{b}\left( {\beta_{R} + \eta } \right)} \right) - \mathop \sum \nolimits_{i = 1}^{n} \alpha_{i} }},\bar{b} = \frac{{\beta_{S} - \mathop \sum \nolimits_{i = 1}^{n} \left( {\alpha_{i} + d_{i} } \right)}}{{\left( {\beta_{S} + \eta } \right)}},1, \ldots ,1} \right)$$.

Equilibrium point *E*_2_ always exists in *Ω*. In addition that, the equilibrium point *E*_3_ is in *Ω*, when $\frac{{\beta_{S} - \mathop \sum \nolimits_{i = 1}^{n} \left( {\alpha_{i} + d_{i} } \right)}}{{\beta_{S} + \eta }} > \frac{{\beta_{R} }}{{\beta_{R} + \eta }}$.

Taking (9) into account, we have that equilibria of system (3) in *Ω* are

13 $$\begin{array}{*{20}l} {E_{0} = \left( {0,0,0,1, \ldots ,1} \right),} \hfill \\ {E_{1} = \left( {0,1,0,1, \ldots ,1} \right),} \hfill \\ {E_{2} = \left( {0,B,B,1, \ldots ,1} \right),} \hfill \\ {E_{3} = \left( {A\frac{A - B}{A - B + C},A\frac{C}{A - B + C},A,1, \ldots ,1} \right),\quad {\text{when}}\,A > B.} \hfill \\ \end{array}$$

### **Theorem 3**

(Routh–Hurwitz Criteria)* Given the polynomial,*

$$P\left( \lambda \right) = \lambda^{n} + a_{1} \lambda^{n - 1} + \cdots + a_{n - 1} \lambda + a_{n} ,$$

*where the coefficients*$a_{i}$* for*$i = 1, \ldots ,n$* are real constants, define then Hurwitz matrices using the coefficients a*_*i*_* of the characteristic polynomial:*

$$H_{1} = \left( {a_{1} } \right),H_{2} = \left( {\begin{array}{*{20}l} {a_{1} } \hfill & 1 \hfill \\ {a_{3} } \hfill & {a_{2} } \hfill \\ \end{array}}\right),H_{3} = \left( {\begin{array}{*{20}l} {a_{1} } \hfill & 1 \hfill & 0 \hfill \\ {a_{3} } \hfill & {a_{2} } \hfill & {a_{1} } \hfill \\ {a_{5} } \hfill & {a_{4} } \hfill & {a_{3} } \hfill \\ \end{array} } \right), \ldots ,H_{n} = \left( {\begin{array}{*{20}l} {a_{1} } \hfill & 1 \hfill & 0 \hfill & 0 \hfill & \cdots \hfill & 0 \hfill \\ {a_{3} } \hfill & {a_{2} } \hfill & {a_{1} } \hfill & 1 \hfill & \cdots \hfill & 0 \hfill \\ {a_{5} } \hfill & {a_{4} } \hfill & {a_{3} } \hfill & {a_{1} } \hfill & \cdots \hfill & 0 \hfill \\ \vdots \hfill & \vdots \hfill & \vdots \hfill & \vdots \hfill & \ddots \hfill & \vdots \hfill \\ 0 \hfill & 0 \hfill & 0 \hfill & 0 \hfill & \cdots \hfill & a \hfill \\ \end{array} } \right)$$

*wherea*_*j*_ = 0* ifj* > *n*.* All of the roots of polynomialP*(*λ*)* are negative or have negative real parts, if and only if the determinants of all Hurwitz matrices are positive: *$det H_{j} > 0, j = 1,2, \ldots ,n$ .* For polynomial of degree*$n = 2, 3, 4$* and* 5,* the Routh–Hurwitz criteria are summarized.*

$$\begin{aligned} & n = 2:a_{1} ,a_{2} > 0, \\ & n = 3:a_{1} ,a_{3} > 0\;\text{and}\;a_{1} a_{2} > a_{3} , \\ & n = 4:a_{1} ,a_{3} ,a_{4} > 0\;\text{and}\;a_{1} a_{2} a_{3} > a_{3}^{2} + a_{1}^{2} a_{4} , \\ & n = 5:a_{1} ,a_{2} ,a_{3} ,a_{4} ,a_{5} > 0,a_{1} a_{2} a_{3} > a_{3}^{2} + a_{1}^{2} a_{4} \;\text{and}\quad\left( {a_{1} a_{4} - a_{5} } \right)\left( {a_{1} a_{2} a_{3} - a_{3}^{2} - a_{1}^{2} a_{4} } \right) > a_{5} \left( {a_{1} a_{2} - a_{3} } \right)^{2} + a_{1} a_{5}^{2} . \\ \end{aligned}$$

This criteria has given necessary and sufficient conditions for all of the roots of the characteristic polynomial (with real coefficients) to lie in the left half of the complex plane (Allen 2007).

### **Theorem 4**

*Suppose*$\frac{dX}{dt} = F\left( X \right)$* is a nonlinear first-order autonomous system with an equilibrium*$\overline{X}$.* Denote the Jacobian matrix ofF evaluated at*$\overline{X}$* as*$J\left( {\overline{X}} \right)$.* If the characteristic equation of the Jacobian matrix*$J\left( {\overline{X}} \right)$,

$$\lambda^{n} + a_{1} \lambda^{n - 1} + a_{2} \lambda^{n - 2} + \cdots + a_{n - 1} \lambda + a_{n} = 0,$$

*satisfies the conditions of the Routh–Hurwitz criteria in Theorem 3, that is, the determinants of all of the Hurwitz matrices are positive, *$det\left( {H_{j} } \right) > 0, j = 1,2, \ldots ,n$,* then the equilibrium*$\overline{X}$* is locally asimptotically stable. If*$det\left( {H_{j} } \right) < 0$,* for some*$j = 1,2, \ldots ,n$,* then the equilibrium*$\overline{X}$* is unstable* (*Allen*^2007^).

The following proposition is shown conditions that equilibrium points in the Proposition 2 are locally asimptotically stability (LAS).

### **Proposition 4**

*The equilibrium points of system* (3)* inΩ satisfy*

(i) *E*_0_* andE*_1_* are unstable points*.
(ii) *IfA* < *B*,* thenE*_2_* is LAS*.
(iii) *LetB* < *A*,* thenE*_3_* is LAS*.

### *Proof*

For the stability analysis, the functions of the right side of system (3) are determined as follows;

14 $$\begin{aligned} \varphi_{1} \left( {s,r,b,a_{i} } \right) &= \beta_{S} s\left( {1 - \left( {s + r} \right)} \right) - \eta sb - s\mathop \sum \limits_{i = 1}^{n} \left( {\alpha_{i} + d_{i} } \right)a_{i} \hfill \\ \varphi_{2} \left( {s,r,b,a_{i} } \right) &= \beta_{R} r\left( {1 - \left( {s + r} \right)} \right) - \eta rb + s\mathop \sum \limits_{i = 1}^{n} \alpha_{i} a_{i} \hfill \\ \varphi_{3} \left( {s,r,b,a_{i} } \right) &= kb\left( {1 - \frac{b}{s + r}} \right) \hfill \\ r_{i} \left( {s,r,b,a_{i} } \right) &= \mu_{i} \left( {1 - a_{i} } \right),\quad i = 1,2, \ldots ,n. \hfill \\ \end{aligned}$$

That jacobian matrix obtained from the equations in (14) is

15 $$J = \left( {\begin{array}{*{20}l} {\left( {\begin{array}{*{20}l} {\beta_{S} - 2\beta_{S} s - } \hfill \\ {\beta_{S} r - \eta b - } \hfill \\ {\mathop \sum \nolimits_{i = 1}^{n} \left( {\alpha_{i} + d_{i} } \right)a_{i} } \hfill \\ \end{array} } \right)} \hfill & { - \beta_{S} s} \hfill & { - \eta s} \hfill & { - s\left( {\alpha_{1} + d_{1} } \right)} \hfill & \cdots \hfill & { - s\left( {\alpha_{n} + d_{n} } \right)} \hfill \\ {\left( {\mathop \sum \nolimits_{i = 1}^{n} \alpha_{i} a_{i} } \right) - \beta_{R} r} \hfill & {\left( {\begin{array}{*{20}l} {\beta_{R} - \beta_{R} s - } \hfill \\ {2\beta_{R} r - \eta b} \hfill \\ \end{array} } \right)} \hfill & { - \eta r} \hfill & {s\alpha_{1} } \hfill & \cdots \hfill & {s\alpha_{n} } \hfill \\ {\frac{{kb^{2} }}{{\left( {s + r} \right)^{2} }}} \hfill & {\frac{{kb^{2} }}{{\left( {s + r} \right)^{2} }}} \hfill & {k - \frac{2kb}{{\left( {s + r} \right)}}} \hfill & 0 \hfill & \cdots \hfill & 0 \hfill \\ 0 \hfill & 0 \hfill & 0 \hfill & { - \mu_{1} } \hfill & \cdots \hfill & 0 \hfill \\ \vdots \hfill & \vdots \hfill & \vdots \hfill & \vdots \hfill & \ddots \hfill & \vdots \hfill \\ 0 \hfill & 0 \hfill & 0 \hfill & 0 \hfill & \cdots \hfill & { - \mu_{n} } \hfill \\ \end{array} } \right)$$

For ease of examination, the *τ*-th eigenvalue of equilibrium point *E*_*k*_ has shown as *λ*_*k*,*τ*_ for *k* = 0, 1, 2, 3 and $\tau = 1,2, \ldots ,n + 3,\quad n \in N$.

(i) Some of the eigenvalues evaluated at the equilibrium point *E*_0_ in *Ω* are $\lambda_{0,1} = \beta_{S} - \sum_{i = 1}^{n} \left( {\alpha_{i} + d_{i} } \right)$ and *λ*_0,2_ = *β*_*R*_. The eigenvalue *λ*_0,2_ is positive, due to (2). According to Theorem 4, the infection-free equilibrium point *E*_0_ is unstable point for system (3).
  In the same way, the eigenvalues for *E*_1_ in *Ω* are that $\lambda_{1,1} = - \sum_{i = 1}^{n} \left( {\alpha_{i} + d_{i} } \right)$, *λ*_1,2_ = −*β*_*R*_, *λ*_1,3_ = *k* and *λ*_1,*i*+3_ = −*μ*_*i*_ for *i* = 1, 2, …, *n*. The eigenvalue *λ*_1,3_ is positive, due to (2). From Theorem 4, *E*_1_ is unstable point for system (3).
(ii) The jacobian matrix in (15) evaluated at the equilibrium point *E*_2_ in *Ω* is given below
  16 $$J\left( {E_{2} } \right) = \left( {\begin{array}{*{20}l} {\beta_{S} - B\left( {\beta_{S} + \eta } \right) - \mathop \sum \nolimits_{i = 1}^{n} \left( {\alpha_{i} + d_{i} } \right)} \hfill & 0 \hfill & 0 \hfill & 0 \hfill & \ldots \hfill & 0 \hfill \\ { - \beta_{R} B + \mathop \sum \nolimits_{i = 1}^{n} \alpha_{i} } \hfill & { - \beta_{R} B} \hfill & { - \eta B} \hfill & 0 \hfill & \ldots \hfill & 0 \hfill \\ k \hfill & k \hfill & { - k} \hfill & 0 \hfill & \ldots \hfill & 0 \hfill \\ 0 \hfill & 0 \hfill & 0 \hfill & { - \mu_{1} } \hfill & \ldots \hfill & 0 \hfill \\ \vdots \hfill & \vdots \hfill & \vdots \hfill & \vdots \hfill & \ddots \hfill & \vdots \hfill \\ 0 \hfill & 0 \hfill & 0 \hfill & 0 \hfill & \ldots \hfill & { - \mu_{n} } \hfill \\ \end{array} } \right).$$
  The eigenvalues of matrix (16) are $\lambda_{2,i + 3} = - \mu_{i}$ for *i* = 1, 2, …, *n* and the remain three eigenvalues are found from following matrix;
  17 $$J^{{B\left( {E_{2} } \right)}} = \left( {\begin{array}{*{20}l} {\beta_{S} - B\left( {\beta_{S} + \eta } \right) - \mathop \sum \nolimits_{i = 1}^{n} \left( {\alpha_{i} + d_{i} } \right)} \hfill &\quad 0 \hfill &\quad 0 \hfill \\ { - \beta_{R} B + \mathop \sum \nolimits_{i = 1}^{n} \alpha_{i} } \hfill &\quad { - \beta_{R} B} \hfill & { - \eta B} \hfill \\ k \hfill &\quad k \hfill &\quad { - k} \hfill \\ \end{array} } \right)$$
  where matrix $J^{{B\left( {E_{2} } \right)}}$ is the block matrix of (16). From (2), it is clear that *Reλ*_2,*i*+3_ < 0. Characteristic equation of (17) is
  18 $$\left[ {\left( {\beta_{S} - B\left( {\beta_{S} + \eta } \right) - \mathop \sum \limits_{i = 1}^{n} \left( {\alpha_{i} + d_{i} } \right)} \right) - \lambda } \right]\left[ {\lambda^{2} + \lambda \left( {k + \beta_{R} B} \right) + k\beta_{R} } \right] = 0.$$
  By the solution of (18), these eigenvalues are determined. Hence, it is obtained that
  19 $$\lambda_{2,1} = \left( {\beta_{S} - B\left( {\beta_{S} + \eta } \right) - \mathop \sum \limits_{i = 1}^{n} \left( {\alpha_{i} + d_{i} } \right)} \right)$$
  and *λ*_2,2_ and *λ*_2,3_ are gained by solving following equation,
  20 $$\lambda^{2} + \lambda \left( {k + \beta_{R} B} \right) + k\beta_{R} = 0.$$
  It can be observed that $\left( {k + \beta_{R} B} \right), k\beta_{R} > 0$, due to (2) and (10). From Theorem 3 (*n* = 2), $Re\lambda_{2,2} \;{\text{and}}\;Re\lambda_{2,3} < 0$. According to Theorem 4, the LAS conditions for *E*_2_ are provided for the eigenvalues, $\lambda_{2,i + 3} , \lambda_{2,2}$ and $\lambda_{2,3}$, exceptionally *λ*_2,1_in the (19). It is sufficient to examine the sign of *λ*_2,1_. By (9), (19) it can be rewrited as *λ*_2,1_ = (*β*_*S*_ + *η*)(*A* − *B*). In this respect, *λ*_2,1_ is negative, when *A* < *B*.
  As a result, the equilibrium point *E*_2_ is LAS for system (3), when *A* < *B*.
(iii) Let *B* < *A*. Then the equilibrium point *E*_3_ is revealed in *Ω*. The Jacobian matrix for *E*_3_ is
  21 $$J\left( {E_{3} } \right) = \left( {\begin{array}{*{20}l} { - \beta_{S} \bar{s}} \hfill & { - \beta_{S} \bar{s}} \hfill & { - \eta \bar{s}} \hfill & { - \bar{s}\left( {\alpha_{1} + d_{1} } \right)} \hfill & \cdots \hfill & { - \bar{s}\left( {\alpha_{n} + d_{n} } \right)} \hfill \\ { - \beta_{R} \bar{r} + \mathop \sum \nolimits_{i = 1}^{n} \alpha_{i} } \hfill & { - \beta_{R} \left( {1 - \bar{b}} \right) - \eta \bar{b} - \beta_{R} \bar{r}} \hfill & { - \eta \bar{r}} \hfill & {\bar{s}\alpha_{1} } \hfill & \cdots \hfill & {\bar{s}\alpha_{n} } \hfill \\ k \hfill & k \hfill & { - k} \hfill & 0 \hfill & \cdots \hfill & 0 \hfill \\ 0 \hfill & 0 \hfill & 0 \hfill & { - \mu_{1} } \hfill & \cdots \hfill & 0 \hfill \\ \vdots \hfill & \vdots \hfill & \vdots \hfill & \vdots \hfill & \ddots \hfill & \vdots \hfill \\ 0 \hfill & 0 \hfill & 0 \hfill & 0 \hfill & \cdots \hfill & { - \mu_{n} } \hfill \\ \end{array} } \right)$$
  where
  22 $$E_{3} = \left( {A\frac{A - B}{A - B + C},A\frac{C}{A - B + C},A,1,1, \ldots ,1} \right) = \left( {\bar{s},\bar{r},\bar{b},1,1, \ldots ,1} \right)\quad {\text{for}}\;A > B.$$
  The eigenvalues obtained from (21) are *λ*_3,*i*+3_ = −*μ*_*i*_ for *i* = 1, 2, …, *n* and the remaining three eigenvalues, *λ*_3,1_, *λ*_3,2_ and *λ*_3,3_, are found from following matrix;
  23 $$J^{{B\left( {E_{3} } \right)}} = \left( {\begin{array}{*{20}l} { - \beta_{S} \bar{s}} \hfill & { - \beta_{S} \bar{s}} \hfill & { - \eta \bar{s}} \hfill \\ { - \beta_{R} \bar{r} + \mathop \sum \nolimits_{i = 1}^{n} \alpha_{i} } \hfill & {\beta_{R} \left( {1 - \bar{b}} \right) - \eta \bar{b} - \beta_{R} \bar{r}} \hfill & { - \eta \bar{r}} \hfill \\ k \hfill & k \hfill & { - k} \hfill \\ \end{array} } \right)$$
  where the matrix $J^{{B\left( {E_{3} } \right)}}$ is the block matrix of (21). By (2), it is *Reλ*_3,*i*+3_ < 0. Characteristic equation of matrix (23) is obtained as follows:
  24 $$\lambda^{3} + a_{1} \lambda^{2} + a_{2} \lambda + a_{3} = 0,$$
  where
  25 $$\begin{array}{*{20}l} {a_{1} = \left( {\left( {\frac{{\bar{s}}}{{\bar{r}}}\mathop \sum \limits_{i = 1}^{n} \alpha_{i} + \beta_{R} \bar{r} + \beta_{S} \bar{s}} \right) + k} \right)} \hfill \\ {a_{2} = \left( {k\left( {\frac{{\bar{s}}}{{\bar{r}}}\mathop \sum \limits_{i = 1}^{n} \alpha_{i} + \beta_{R} \bar{r} + \beta_{S} \bar{s}} \right) + \bar{b}\eta k + \beta_{S} \bar{b}\frac{{\bar{s}}}{{\bar{r}}}\mathop \sum \limits_{i = 1}^{n} \alpha_{i} } \right)} \hfill \\ {a_{3} = k\eta \bar{b}\frac{{\bar{s}}}{{\bar{r}}}\mathop \sum \limits_{i = 1}^{n} \alpha_{i} + k\beta_{S} \bar{b}\frac{{\bar{s}}}{{\bar{r}}}\mathop \sum \limits_{i = 1}^{n} \alpha_{i} .} \hfill \\ \end{array}$$
  We have *a*_1_, *a*_3_ > 0 due to (2) and (22). By (25), we have that
  $$\begin{aligned} a_{1} a_{2} - a_{3} & = \left( {\left( {\frac{{\bar{s}}}{{\bar{r}}}\sum\limits_{{i = 1}}^{n} {\alpha _{i} } + \beta _{R} \bar{r} + \beta _{S} \bar{s}} \right) + k} \right)\left( {k\left( {\frac{{\bar{s}}}{{\bar{r}}}\sum\limits_{{i = 1}}^{n} {\alpha _{i} } + \beta _{R} \bar{r} + \beta _{S} \bar{s}} \right) + \bar{b}\eta k + \bar{b}\beta _{S} \frac{{\bar{s}}}{{\bar{r}}}\sum\limits_{{i = 1}}^{n} {\alpha _{i} } } \right) \\ & \quad - k\bar{b}\left( {\frac{{\bar{s}}}{{\bar{r}}}\sum\limits_{{i = 1}}^{n} {\alpha _{i} } } \right)\left( {\eta + \beta _{S} } \right), \\ \end{aligned}$$
  $$\begin{aligned}a_{1} a_{2} - a_{3} =&\, \left( {\frac{{\bar{s}}}{{\bar{r}}}\mathop \sum \limits_{i = 1}^{n} \alpha_{i} } \right)\bar{b}\eta k + \left({\frac{{\bar{s}}}{{\bar{r}}}\mathop \sum \limits_{i = 1}^{n} \alpha_{i} } \right)\left( {\bar{b}\beta_{S} \left( {\frac{{\bar{s}}}{{\bar{r}}}\mathop \sum \limits_{i = 1}^{n} \alpha_{i} } \right) + k\left( {\frac{{\bar{s}}}{{\bar{r}}}\mathop \sum \limits_{i = 1}^{n} \alpha_{i} + \beta_{R} \bar{r} + \beta_{S} \bar{s}} \right)} \right) \\ &+ k\bar{b}\beta_{S} \left( {\frac{{\bar{s}}}{{\bar{r}}}\mathop \sum \limits_{i = 1}^{n} \alpha_{i} } \right) + k\left( {\bar{b}\eta k + k\left( {\frac{{\bar{s}}}{{\bar{r}}}\mathop \sum \limits_{i = 1}^{n} \alpha_{i} + \beta_{R} \bar{r} + \beta_{S} \bar{s}} \right)} \right)\\ &+ \left( {\beta_{R} \bar{r} + \beta_{S} \bar{s}} \right)\left( {\bar{b}\eta k + \bar{b}\beta_{S} \left( {\frac{{\bar{s}}}{{\bar{r}}}\mathop \sum \limits_{i = 1}^{n}\alpha_{i} } \right) + k\left( {\frac{{\bar{s}}}{{\bar{r}}}\mathop\sum \limits_{i = 1}^{n} \alpha_{i} + \beta_{R} \bar{r} + \beta_{S}\bar{s}} \right)} \right) \\ & - k\bar{b}\left({\frac{{\bar{s}}}{{\bar{r}}}\mathop \sum \limits_{i = 1}^{n}\alpha_{i} } \right)\left( {\eta + \beta_{S} } \right)\end{aligned}$$
  and so,
  $$\begin{aligned} a_{1} a_{2} - a_{3} &= \left( {\frac{{\bar{s}}}{{\bar{r}}}\mathop \sum \limits_{i = 1}^{n} \alpha_{i} } \right)\left( {\bar{b}\beta_{S} \left( {\frac{{\bar{s}}}{{\bar{r}}}\mathop \sum \limits_{i = 1}^{n} \alpha_{i} } \right) + k\left( {\frac{{\bar{s}}}{{\bar{r}}}\mathop \sum \limits_{i = 1}^{n} \alpha_{i} + \beta_{R} \bar{r} + \beta_{S} \bar{s}} \right)} \right) \\&\quad+ k\left( {\bar{b}\eta k + k\left( {\frac{{\bar{s}}}{{\bar{r}}}\mathop \sum \limits_{i = 1}^{n} \alpha_{i} + \beta_{R} \bar{r} + \beta_{S} \bar{s}} \right)} \right) \\ &\quad+ \left( {\beta_{R} \bar{r} + \beta_{S} \bar{s}} \right)\left( {\bar{b}\eta k + \bar{b}\beta_{S} \left( {\frac{{\bar{s}}}{{\bar{r}}}\mathop \sum \limits_{i = 1}^{n} \alpha_{i} } \right) + k\left( {\frac{{\bar{s}}}{{\bar{r}}}\mathop \sum \limits_{i = 1}^{n} \alpha_{i} + \beta_{R} \bar{r} + \beta_{S} \bar{s}} \right)} \right) \\ &\quad\underbrace {{ + \left( {\frac{{\bar{s}}}{{\bar{r}}}\mathop \sum \limits_{i = 1}^{n} \alpha_{i} } \right)\bar{b}\eta k + k\bar{b}\beta_{S} \left( {\frac{{\bar{s}}}{{\bar{r}}}\mathop \sum \limits_{i = 1}^{n} \alpha_{i} } \right) - k\bar{b}\left( {\frac{{\bar{s}}}{{\bar{r}}}\mathop \sum \limits_{i = 1}^{n} \alpha_{i} } \right)\left( {\eta + \beta_{S} } \right).}}_{ = 0} \hfill \\ \end{aligned}$$
  Therefore, it is obtained that *a*_1_*a*_2_ − *a*_3_ > 0 due to (2) and (22). From Theorem 3(*n* = 3), *Reλ*_3,1_, *Reλ*_3,2_ and *Reλ*_3,3_ < 0. According to Theorem 4, The equilibrium point *E*_3_ in *Ω* is LAS for the system (3), when it exists biological, that is, *B* < *A*. Hence, proof is completed.

The LAS conditions found for equilibrium points in (13) are summarized in the Table 1.

**Table 1:** Existence and stability conditions of the equilibria of system (3)

Equilibrium points	Biological existence conditions	LAS conditions
$E_{0} = \left( {0,0,0,1, \ldots ,1} \right)$	Always exists	Unstable
$E_{1} = \left( {0,1,0,1, \ldots ,1} \right)$	Always exists	Unstable
$E_{2} = \left( {0,B,B,1, \ldots ,1} \right)$	Always exists	$A < B$
$E_{3} = \left( {A\frac{A - B}{A - B + C},A\frac{C}{A - B + C},A,1,1, \ldots ,1} \right)$	$B < A$	When it exists biological

Where the values $A$, $B$ and $C$ are as indicated in (9)

### **Proposition 6**

*Let us denote by Γ*_2 _*the LAS region of the equilibrium pointE*_2_*inΩ*. *In the same way,Γ*_3_* is forE*_3_.* ThenΓ*_2_ ∩ *Γ*_3_ = ∅.

### *Proof*

It can be clearly observed in Table 1.

The parameter values and it’s references used for numerical study are given in Table 2.

**Table 2:** Interpretation and considered values of the parameters

Parameter	Description	Value	References
$\beta_{S}$	Growth rate of sensitive bacteria	0.8 day^−1^	Mondragón et al. (2014)
$( {1 - c} )\beta_{S}, \,0 < c < 1$	Growth rate of resistant bacteria	0.4–0.1 day^−1^	Mondragón et al. (2014)-hypothesis
$k$	Growth rate of immune cells	0.6 day^−1^	Pugliese and Gandolfi (2008)
$\eta$	Rate of bacteria destroyed by immune cells	0.3 day^−1^	Pugliese and Gandolfi (2008)
$\omega$	Rate to the amount of present bacteria of carrying capacity of immune cells	1	Hypothesis
$T$	Carrying capacity of bacteria	10^9^ bacteria	Alavez et al. (2006)
$\overline{{\alpha_{1} }}$	Mutation rate of INH	10^−6^ mut×gen	Coll (2009)
$\overline{{\alpha_{2} }}$	Mutation rate of PZA	0	Mondragón et al. (2014)
$\overline{{d_{1} }}$	Elimination rate of sensitive bacteria due INH	0.0039 day^−1^	Zhang (2009)
$\overline{{d_{2} }}$	Elimination rate of sensitive bacteria due PZA	0.0001625 day^−1^	Alavez et al. (2006)
$\delta_{1}$	Daily dose of INH	5 mg/kg/day	Coll (2009)
$\delta_{2}$	Daily dose of ZPA	35–20 mg/kg/day	Coll (2009)
$\mu_{1}$	Uptake rate of INH	0.06 day^−1^	Esteva et al. (2011)
$\mu_{2}$	Uptake rate of PZA	0.03 day^−1^	Esteva et al. (2011)

Datas are deduced from the literature

The values obtained from this table are that (i) in the first case, $A = 0.260674$, $B = 0.571428$ $\left( {A < B} \right)$ and so, $E_{2} = \left( {0,0.5714,0.5714,1, \ldots ,1} \right)$ is LAS. (ii) in the second case, $A = 0.334538$, $B = 0.25$ $\left( {A > B} \right)$ and so, $E_{3} = \left( {0.3337,0.0008,0.3345,1,1, \ldots ,1} \right)$ is LAS

Among the treatment regimen recommended by WHO includes isoniazid (INH) and pyrazinamide (PZA) for some bacterial infectious (such as *Mycobacterium tuberculosis*) (Coll 2009)

### **Proposition 7**

*IfA* < *B*,* then the equilibrium pointE*_2_ = (0, *B*, *B*, 1, …, 1)* is globally asimptotically stable (GAS) inΩ*.

### *Proof*

Their solutions approach *a*_*i*_ = 1 for *i* = 1, 2, …, *n*, when last *n* equations of system (3) are considered separately. Replacing *s* = 0 and *a*_*i*_ = 1 for *i* = 1, 2, …, *n* in system (3), we attain the asymptotically equivalent planar system (Mondragón et al. 2014) in the region *Ω*_1_ = {(*r*, *b*) ∊ *R*^2^:0 < *b* ≤ *r* ≤ 1} given by

26 $$\begin{array}{*{20}l} {\frac{dr}{dt} = f\left( {r,b} \right) = \beta_{R} r\left( {1 - r} \right) - \eta rb} \hfill \\ {\frac{db}{dt} = g\left( {r,b} \right) = kb\left( {1 - \frac{b}{r}} \right)} \hfill \\ \end{array}$$

According to the Dulac criterion, there exists a continuously differentiable Dulac function *Φ*(*r*, *b*) for a simply connected region *Ω*_1_ ⊂ *R*^2^ such that

27 $$\varPhi \left( {r,b} \right) = \frac{1}{rb}.$$

Since (*f*(*r*, *b*), *g*(*r*, *b*)) is the vector field of system (26),

$$\frac{{\partial \left[ {\varPhi \left( {r,b} \right)f\left( {r,b} \right)} \right]}}{\partial r} + \frac{{\partial \left[ {\varPhi \left( {r,b} \right)g\left( {r,b} \right)} \right]}}{\partial b} = \frac{\partial }{\partial r}\left[ {\frac{{\beta_{R} \left( {1 - r} \right) - \eta b}}{b}} \right] + \frac{\partial }{\partial b}\left[ {\frac{{k\left( {1 - \frac{b}{r}} \right)}}{r}} \right] = - \left( {\frac{{\beta_{R} }}{b} + \frac{k}{{r^{2} }}} \right) < 0.$$

This result reveals that system (26) has no periodic orbits contained in the interior of *Ω*_1_ in compliance with Dulac-Bendixon criterion. In addition, the region *Γ*_2_ cited in the Proposition 6 does not include another the LAS equilibrium point. Thus, by the Poincaré–Bendixon Theorem and Dulac–Bendixon criterion, we have that equilibrium point *E*_2_ = (0, *B*, *B*, 1, …, 1) is GAS.

**Fig. 1:**
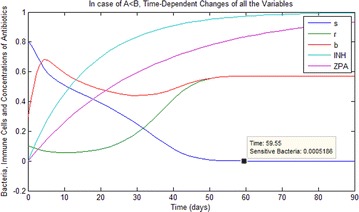
In case of (i) in the Table 2, time-dependent changes of all the variables

**Fig. 2:**
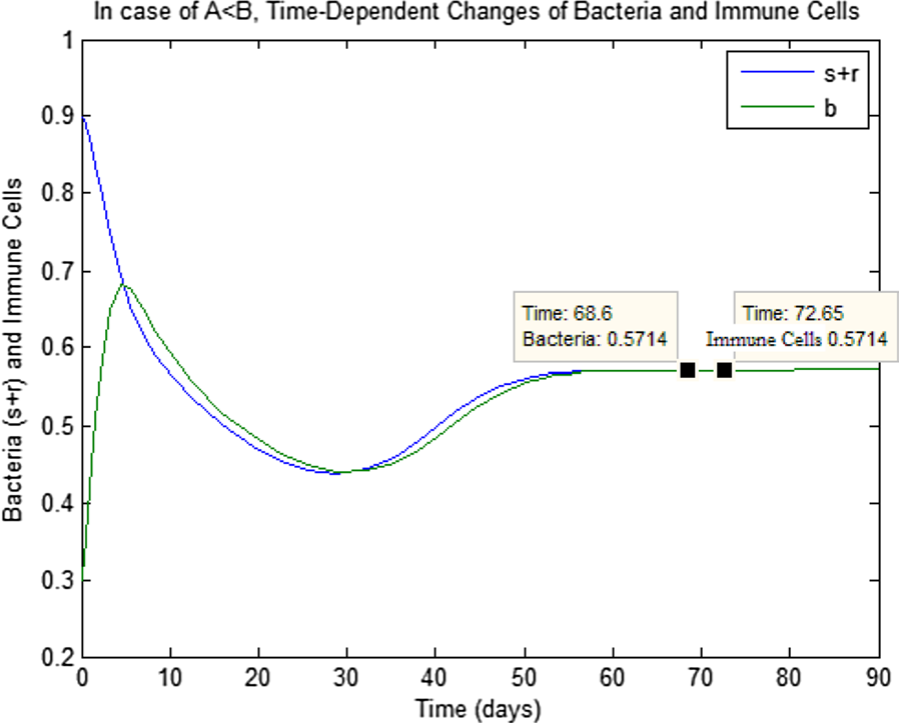
In case of (i) in the Table 2, time-dependent changes of bacteria and immune cells

**Fig. 3:**
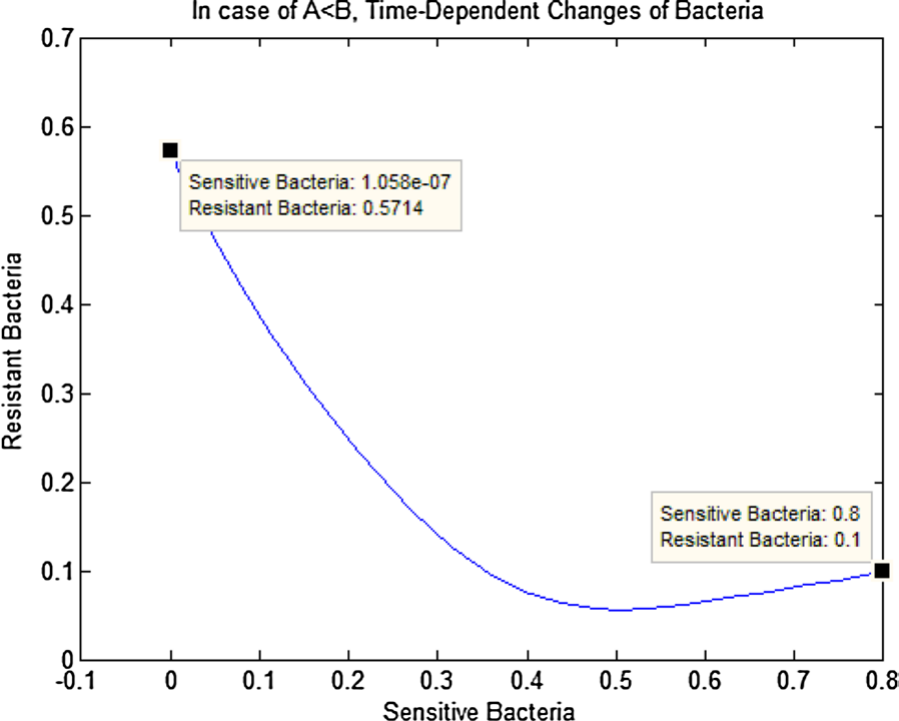
In case of (i) in the Table 2, time-dependent changes of bacteria

For status (i) obtained from the Table 2, the qualitative analysis of the system (2) has supported by numerical simulations in the following Figs. 1, 2 and 3. Here, it is shown that the equilibrium point *E*_2_ is GAS. Also, as a result of using multiple antibiotics at least for 60 days, sensitive bacteria is removed and resistant bacteria and immune cells have same equilibria value of $\frac{{\beta_{R} }}{{\beta_{R} + \eta }}$.

### **Proposition 8**

*LetB* < *A*.* Then,*

28 $$\frac{{b - \bar{b}}}{b}\frac{db}{dt} < 0$$

*where the values*$\bar{s},\bar{r}$* and *$\bar{b}$* are in* (22).

### *Proof*

Let $b < \bar{b}$. This shows that *b* is an increasing function and approach asymptotically to $\bar{b}$ by increasing in *Ω*. From the third equation in system (3), it is obtained that $\frac{db}{dt} = kb\left( {1 - \frac{b}{s + r}} \right) > 0$ except for $\bar{b} = \bar{s} + \bar{r}$ (state of equilibrium). In this sense, we have that $\frac{{b - \bar{b}}}{b}\frac{db}{dt} < 0$.

Let $b > \bar{b}$. In the same way, *b* is approaches asymptotically to $\bar{b}$ by decreasing. Thus, it is obtained that $\frac{{b - \bar{b}}}{b}\frac{db}{dt} < 0$. Proposition is proved.

### **Proposition 9**

*LetA* > *B*.* If*$1 - \bar{r} < s$* and*$\frac{{A - \left( {1 - {{\sigma }}} \right)b}}{{{\sigma }}} < s + r < \frac{{A - \left( {1 - B} \right)b}}{B}$,* then equilibrium point*$E_{3}$* is GAS in the region*

29 $$\varOmega_{2} = \left\{ {\left( {s,r,b} \right) \in R^{3} :0 < b \le s + r \le 1} \right\}.$$

*where*$$\sigma = \frac{{\beta_{S} }}{{\beta_{S} + \eta }}$$.

### *Proof*

If the last $n$ equations of system (3) are separated, then their solutions approach $a_{i} = 1$ for $i = 1,2, \ldots ,n$. Replacing these values in the first three equations of system (3), we attain the following:

30 $$\begin{array}{*{20}l} {\frac{ds}{dt} = \beta_{S} s\left( {1 - \left( {s + r} \right)} \right) - \eta sb - s\mathop \sum \limits_{i = 1}^{n} \left( {\alpha_{i} + d_{i} } \right)} \hfill \\ {\frac{dr}{dt} = \beta_{R} r\left( {1 - \left( {s + r} \right)} \right) - \eta rb + s\mathop \sum \limits_{i = 1}^{n} \alpha_{i} } \hfill \\ {\frac{db}{dt} = kb\left( {1 - \frac{b}{s + r}} \right).} \hfill \\ \end{array}$$

in the region (29). Considering (9), then the system (30) transforms to

31 $$\begin{array}{*{20}l} {\frac{ds}{dt} = \left( {\beta_{S} + \eta } \right)s\left( {A - \left( {s + r} \right)\sigma - b\left( {1 - \sigma } \right)} \right)} \hfill \\ {\frac{dr}{dt} = \left( {\beta_{R} + \eta } \right)\left( {r\left( {1 - \left( {s + r} \right)} \right)B - rb\left( {1 - B} \right) + sC} \right)} \hfill \\ {\frac{db}{dt} = kb\left( {1 - \frac{b}{s + r}} \right)} \hfill \\ \end{array}$$

where $\sigma = \frac{{\beta_{S} }}{{\beta_{S} + \eta }}$. Also, it is clear that $\sigma > A$.

In this context, the GAS of equilibrium point $E_{3}$ can be obtained by applying the LaSalle-Lyapunov Theorem. Lyapunov function is

32 $$V\left( {s,r,b} \right) = \left( {c_{1} \left( {s - \bar{s} - \bar{s}{ \ln }\frac{s}{{\bar{s}}}} \right) + c_{2} \left( {r - \bar{r} - \bar{r}{ \ln }\frac{r}{{\bar{r}}}} \right) + c_{3} \left( {b - \bar{b} - \bar{b}{ \ln }\frac{b}{{\bar{b}}}} \right)} \right)$$

where $c_{1}$, $c_{2}$ and $c_{3}$ are arbitrary positive constants and $\bar{s}$, $\bar{r}$ and $\bar{b}$ are in (22). Differentiating $V\left( {s,r,b} \right)$ with respect to $t$, we get

33 $$\frac{dV}{dt} = c_{1} \frac{{s - \bar{s}}}{s}\frac{ds}{dt} + c_{2} \frac{{r - \bar{r}}}{r}\frac{dr}{dt} + c_{3} \frac{{b - \bar{b}}}{b}\frac{db}{dt}$$

Let $1 - \bar{r} < s$. In this case, since $0 < 1 - \left( {\bar{s} + \bar{r}} \right) < s - \bar{s}$ and $1 - \bar{r} + r < s + r < 1$, it is obtained that

34 $$\bar{s}< {s},\quad \bar{r} > r,$$

respectively. In addition, if $\frac{{A - \left( {1 - {{\sigma }}} \right)b}}{{{\sigma }}} < \left( {s + r} \right) < \frac{{B - \left( {1 - B} \right)b}}{B}$, then

35 $$\frac{ds}{dt}< {0},\quad\frac{dr}{dt} > 0.$$

By (34) and (35), we have

36 $$\frac{{s - \bar{s}}}{s}\frac{ds}{dt} < 0,\quad \frac{{r - \bar{r}}}{r}\frac{dr}{dt} < 0.$$

From (28) and (36), It is obtained that $\frac{dV}{dt} < 0$. In this respect, if $1 - \bar{r} < s$ and $\frac{{A - \left( {1 - {{\sigma }}} \right)b}}{{{\sigma }}} < s + r < \frac{{B - \left( {1 - B} \right)b}}{B}$, then equilibrium point $E_{3}$ is GAS for system (31) in $\varOmega_{2}$, and so, for system (3) in $\varOmega$. Proposition is proved.

**Fig. 4:**
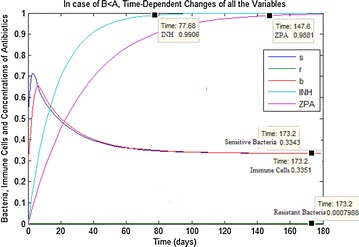
In case of (ii) in the Table 2, time-dependent changes of all the variables

For status (ii) obtained from the Table 2, the qualitative analysis of the system (3) has supported by numerical simulations in Fig. 4. In this case, it is found that $E_{3}$ is GAS. Moreover, sensitive and resistant bacteria to multiple antibiotics and immune system cells have positive equilibrium values as in $E_{3}$.

## Conclusions

In this paper, we formulated a mathematical model of bacterial resistance to immune system response and multiple antibiotics simultaneously, considering specific changes in bacterial DNA sequence as the only mechanism of bacterial resistance acquisition in order to evaluate the effectiveness of antibiotic treatments with respect to the mechanism above.

The parameter $A$ is interpreted as the number of bacteria produced by the fraction of sensitive bacteria that survive to the effects due to antibiotics and immune cells. Similarly, the parameter $B$ represents the bacteria produced by resistant bacteria. Also, the parameter $\eta$ is expresses as effect of immune cells on the bacteria .

Our model is quite appropriate when compared to the complexity of biological phenomenon and it predicts in terms of the parameters $A$ and $B$ when the bacterial progression is either for resistant bacteria and immune cellsas shown in Figs. 1, 2 and 3 or for sensitive and resistant bacteria and immune cells as shown in Fig. 4.

The model suggests that if sensitive bacteria can infect but do not produce sufficient progeny (in case of $A < B$) then they can be removed and resistant bacteria continue to survive in balance with the immune cells in the host. When sensitive bacteria persist, the model predicts the scenario that the immune response of host and antibiotics is not enough to eliminate them (in case of $A > B$), and therefore both types of bacteria continue to survive and coexist in balance with the immune cells in the host.

According to the results of this analysis, the infection never disappears. Also, the infection is continued by resistant bacteria, when the appropriate antibiotics are used, otherwise sensitive and resistant bacteria. The magnitude of infection depends on the effect of immune system in the first case and multiple antibiotics and immune system in the second case.

These results in our model highlight the fact that those whose immunity response against infections have diminished, suffer from the same bacterial infections more. Furthermore, this model shows that some of the bacterial infections believed to be limited or destroyed, make an individual whose immune system deteriorated suffer more.

Additionally, the results obtained from numerical studies in terms of bacterial infection reveal the affinity substantially with reference to the clinical treatment.

For future work we are planing consider other mechanisms such as the loss of resistance in the resistant bacteria and gaining resistance by conjugation of sensitive and resistant bacteria in order to get more accurate results.
